# The Role of Endoscopic Ultrasound-Guided Shear Wave Elastography in Pancreatic Diseases

**DOI:** 10.3390/diagnostics14202329

**Published:** 2024-10-19

**Authors:** Yazan Abboud, Srinivas Gaddam

**Affiliations:** 1 Department of Internal Medicine, Rutgers New Jersey Medical School, Newark, NJ 07103, USA; yazanabboud.md@gmail.com; 2 Karsh Division of Gastroenterology and Hepatology, Cedars-Sinai Medical Center, Los Angeles, CA 90048, USA

**Keywords:** endoscopic ultrasound, shear wave elastography, chronic pancreatitis, pancreatic tumors, pancreatic cancer, fatty pancreas

## Abstract

Elastography is a non-invasive imaging modality that has been developed for the evaluation of the stiffness of various organs. It is categorized into two main types: strain elastography and shear wave elastography. While strain elastography offers valuable information on the mechanical properties of the organ being studied, it is limited by the qualitative nature of its measurements and its reliance on operator skills. On the other hand, shear wave elastography overcomes these limitations as it provides a quantitative assessment of tissue stiffness, offers more reproducibility, and is less operator-dependent. Endoscopic ultrasound-guided shear wave elastography (EUS-SWE) is an emerging technique that overcomes the limitations of transabdominal ultrasound in the evaluation of the pancreas. A growing body of literature has demonstrated its safety and feasibility in the evaluation of pancreatic parenchyma. This article provides a comprehensive review of the current state of the literature on EUS-SWE, including its technical aspects, clinical applications in the evaluation of various pancreatic conditions, technological limitations, and future directions.

## 1. Background

**Figure 1:**
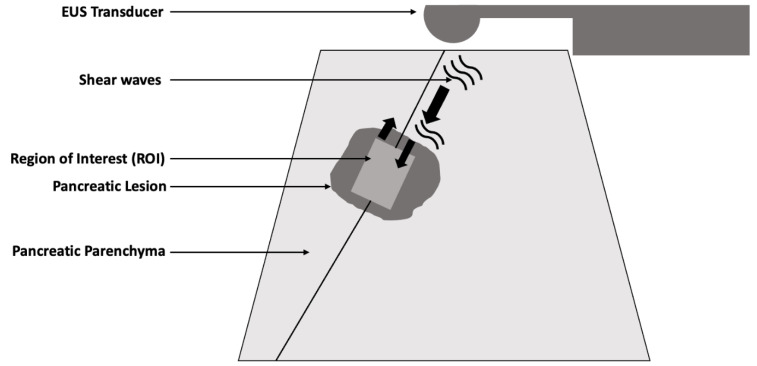
Shear wave elastography method using endoscopic ultrasound where the endoscopic ultrasound transducer generates an acoustic radiation force which propagates through the pancreatic tissue.

Elastography is a non-invasive imaging technology that has been widely studied in the evaluation of various organs including the liver, thyroid, and breast [1,2,3,4]. It evaluates the mechanical properties of tissues, focusing on assessing their stiffness [5]. Evaluation of tissue stiffness is essential in diagnosing and monitoring conditions such as fibrosis, given its impact on organ function [6]. For instance, elastography is often the preferred modality of assessment in evaluating metabolic dysfunction-associated steatotic liver disease (MASLD) as it provides valuable data on the degree of liver fibrosis. Elastography modalities include two main categories: strain elastography (SE) and shear wave elastography (SWE). Each of these methods provides distinct types of information about tissue stiffness. Strain elastography operates by applying external compression pressure to the tissue. It provides qualitative data, which can be visualized as color-coded images that represent levels of tissue stiffness [7]. While strain elastography can provide useful data, it is limited by its reliance on the operator’s skill and the external pressure applied to the tissue being studied [8]. A study by Dong et al. prospectively evaluated the inter-observer reproducibility of strain elastography between two operators when used in the evaluation of 124 breast lesions. The authors showed moderate inter-observer reproducibility when measuring the tissue elasticity value, suggesting that different operators might obtain varying results when performing strain elastography. In contrast, in SWE, the endoscopic ultrasound probe generates acoustic radiation force (“push pulse”), which produces shear waves that propagate through the tissue (Figure 1) [9,10,11]. The velocity of the propagated shear waves through the tissues is measured to provide a quantitative assessment of tissue stiffness. Furthermore, prior literature showed that SWE tends to be more reproducible compared to compression elastography, a type of strain elastography [12]. Thus, SWE is generally more reproducible and less operator-dependent compared to strain elastography [13].

The utilization of transabdominal elastography in the evaluation of pancreatic disease is limited by the deep anatomical location of the pancreas. This deep positioning makes it difficult for the transabdominal elastography probe to effectively visualize and assess the pancreas. This is especially challenging in patients who are obese, pregnant, or have significant bowel gas, which further hinders the probe’s ability to capture accurate measurements. To address these limitations, several studies have explored the potential of elastography when performed with endoscopic ultrasound (EUS) [14,15]. EUS offers a closer and more direct approach to the pancreas, potentially overcoming the obstacles faced by transabdominal techniques, and providing more reliable assessments of pancreatic tissue stiffness. A prospective study conducted by Okasha et al., involving 325 patients known to have solid pancreatic lesions on prior imaging at a single center between 2014 and 2017, compared EUS-guided strain elastography (EUS-SE) measurements to pathological examination from EUS-guided fine needle aspiration (FNA). The study showed that EUS-SE, utilizing elastography measurements with strain ratio, had an overall accuracy of 89% with a sensitivity of 97% and specificity of 63% in differentiating malignant from benign pancreatic lesions when considering the FNA-based histopathological examination as the standard diagnostic method [16]. Additionally, a study by Iglesias-Garcia et al. prospectively evaluated the role of EUS-SE in 191 patients who underwent EUS for epigastric pain or chronic pancreatitis over a one-year period. The authors showed that EUS-SE measurements had an accuracy of 91.1% with a sensitivity of 91.2% and a specificity of 91% in the diagnosis of chronic pancreatitis, when compared to the Rosemont criteria as the standard diagnostic method [17].

Despite these results, there remain several limitations that hinder the wide acceptance of EUS-SE in the evaluation of pancreatic disease. Strain elastography is not able to measure absolute tissue stiffness; it measures the elasticity of tissue relative to nearby structures rather than providing a direct measurement of the tissue’s absolute elasticity [18]. Furthermore, it usually provides qualitative results in color-coded images rather than quantitative data [19]. It is also operator-dependent and may not be able to penetrate deeper structures. SWE, on the other hand, measures direct tissue stiffness and provides quantitative data, which can be more reproducible compared to quantitative or semi-quantitative measures of SE [20,21]. When performing EUS-SE of soft tissue, such as the pancreas, the coupling of the transducer, which is required for high-quality EUS images, can alter the amount of pressure on the tissue, influencing the stiffness and thus leading to erroneous readings. Thus, SWE has been shown to have high intra- and inter-observer reproducibility [22]. Mulabecirovic et al. conducted an in vitro study in which they evaluated strain and shear wave elastography modalities and found that shear wave elastography had good to excellent reproducibility [22]. SWE is also less operator-dependent and can penetrate deeper into the tissues [23]. A prior study showed that variations in the orientation of the ROI or the amount of pressure applied by the endoscopist on the transducer did not impact the accuracy of the measurements [24]. Thus, SWE is the preferred modality for measuring stiffness in soft organs such as the breast and thyroid [25,26,27].

As a result of the strengths of the EUS approach and SWE technique compared to the transabdominal approach and SE technique, several studies have evaluated the feasibility of EUS-guided SWE (EUS-SWE) in assessing pancreatic tissue stiffness. The initial feasibility study on EUS-guided SWE was conducted in Japan and published in 2019 [28]. Ohno et al. conducted a prospective evaluation on 42 patients who underwent standard-of-care EUS to evaluate pancreaticobiliary diseases between 2017 and 2018 at a single center. The study reported a total of 1102 EUS-SWE measurements in the head, body, and tail of the pancreas, without any perioperative adverse events, and a 96.8% measurement success rate [28]. Building on this, Abboud et al. published the first experience on EUS-SWE in the United States in 2023 [10]. In this study, a prospective evaluation of a large single-center cohort, including 117 patients who underwent clinically warranted EUS, was conducted. During the study period between 2021 and 2022, five endoscopists collectively performed a total of 3320 EUS-SWE measurements. This demonstrated that EUS-SWE was safe, with no peri-procedural complications, and was feasible with 100% success in obtaining readings from the pancreas. The authors also reported intraclass correlation coefficient (ICC) analysis demonstrating that EUS-SWE is highly reproducible for the evaluation of the pancreas across all three anatomical locations (head, body, and tail of the pancreas) [10]. The greatest reproducibility was found in the body of the pancreas (ICC 0.89, 95% CI 0.81–0.94), followed by the head (ICC 0.85, 95% CI 0.75–0.91) and tail (ICC 0.84, 95% CI 0.69–0.92).

EUS-SWE is still relatively novel, and experience with the technique is limited. Wang et al. conducted a benchtop study in 2023, in which they investigated EUS-SWE using an in vivo porcine model. Their study focused on standardizing ROI measurements, aiming to establish consistent protocols and improve the accuracy and reproducibility of EUS-SWE in clinical practice [24]. They reported that the most accurate measurements were obtained using an ROI with dimensions of 15 mm in height, 10 mm in width, and 10 mm in depth. They demonstrated that the consistency and reliability of EUS-SWE measurements are robust against changes in the orientation of the ROI and the pressure applied on the transducer. It is also recommended that EUS-SWE measurements be performed during periods of minimal respiratory movement to minimize breathing artifacts [9]. Endoscopists at different centers have utilized this technique in prior studies to enhance the precision and reliability of EUS-SWE [10,28]. This practice helps ensure more precise and reliable results, as respiratory motion can introduce variability and distort the measurements [29].

## 2. Technical Aspects of Endoscopic Ultrasound-Guided Shear Wave Elastography

When performing EUS-SWE measurements, the endo-sonographer identifies a region of interest (ROI) on the desired pancreatic parenchyma, while trying to avoid adjacent blood vessels, biliary or pancreatic ducts, or cysts. Typically, the ROI dimensions measure 5 mm, 10 mm, or 15 mm in height and 10 mm in width, with variations in depth depending on the specific anatomical location. With the ROI placed on the pancreas during an EUS exam, SWE measurements can be obtained. The number of measurements and techniques of evaluation may differ based on the manufacturer. Currently, the Hitachi processor, Arietta 850, or its equivalent model is used by both Olympus (Olympus Co., Shinjuku City, Tokyo, Japan) and Fujinon (Fujifilm Co., Minato City, Tokyo, Japan) at the time of writing this article.

SWE Velocity (Vs) is the speed at which the shear waves propagate through the tissue. It is measured by calculating the square root of the quotient obtained by dividing the shear modulus (*G*) by the density of the tissue (*p*) [30,31]. Vs measurements (meters/second “m/s”) are obtained within a designated ROI in the pancreas. These measurements are processed and calculated by the software integrated into the EUS machine. For each measurement, multiple Vs measurements are taken, and the reported Vs value is the median of these measurements that were obtained at a certain ROI. Each final Vs measurement has an interquartile range (IQR) which provides information on the variation between the multiple Vs measurements used to generate the final Vs value. The relationship between tissue stiffness and SWE Vs is direct; stiffer tissues exhibit higher elasticity and therefore higher Vs. Usually, ten Vs measurements are obtained at each ROI with ten associated IQR values [10].

**Figure 2:**
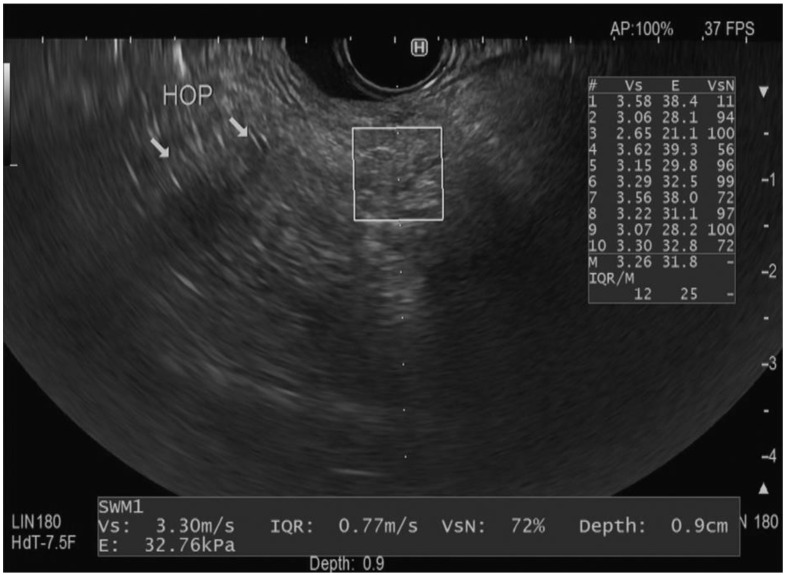
Endoscopic ultrasound view of the shear wave elastography technique in a region of interest (square) in the head of the pancreas with measurements including the velocity (Vs), interquartile range (IQR), reliability (VsN), elasticity (E), and depth [10]. The arrows reflect the pancreatic parenchyma in the head of the pancreas.

Each Vs measurement is accompanied by a reliability index (VsN). This index is expressed as a percentage and indicates the reliability of the measurement. The VsN helps in assessing whether external factors other than the shear wave velocity, such as artifacts or technical issues, might have influenced the reading. Prior research has established that VsN > 50% generally implies a reliable Vs measurement [10,32]. Furthermore, the software also provides another parameter, which is the Elasticity (E) of the tissue (measured by kilopascals “kp”), which represents the tissue’s stiffness [12]. It is measured by the Young module *E* = 3(Vs^2^ρ) [33]. By evaluating the median Vs measurement with its associated IQR and elasticity, clinicians can gain comprehensive insights into the mechanical properties and degree of stiffness/fibrosis of the pancreatic parenchyma. Figure 2 represents an example of EUS-SWE measurement in the head of the pancreas.

## 3. Clinical Applications of Endoscopic Ultrasound-Guided Shear Wave Elastography

Since the initial feasibility studies on EUS-SWE were published, there has been an expanding body of literature exploring its applications in the evaluation of various pancreatic diseases. This includes evaluating the role of EUS-SWE in pancreatic tumors, acute and chronic pancreatitis, and fatty pancreas.

### 3.1. Pancreatic Tumors

The utilization of EUS-SWE for evaluating pancreatic tumors has been increasingly explored in recent years. A retrospective study by Ohno et al. between 2017 and 2019, analyzing prospectively collected data, included 64 patients and examined the role of EUS-SWE in differentiating pancreatic tumors [34]. The authors showed that the median Vs values for pancreatic cancer, pancreatic neuroendocrine tumors, mass-forming pancreatitis, and metastatic tumors were 2.19 m/s, 1.31 m/s, 2.56 m/s, and 1.58 m/s, respectively. The study reported that the Vs measurements using EUS-SWE did not significantly differ between these pancreatic pathologies, suggesting that shear wave velocity may not be sufficient to differentiate between them [34]. To the best of our knowledge, this is the only study evaluating EUS-SWE in pancreatic neuroendocrine tumors. The authors also showed that there was no difference in elasticity between pancreatic cancer and mass-forming pancreatitis. In contrast, the study also examined conventional EUS-SE in the same cohort and noted that the mean strain values for pancreatic cancer, pancreatic neuroendocrine tumors, and mass-forming pancreatitis were 45.4, 47.3, and 74.5, respectively. The study demonstrated that the mean strain value for pancreatic cancer was notably lower compared to other conditions (*p* < 0.001). This study suggested that conventional EUS-SE may offer superior diagnostic performance compared to EUS-SWE in differentiating pancreatic tumors. However, the study does not specify the histopathological subtype of pancreatic cancer, and these findings will need future external validation. The study by Ohno et al. [34]. provides valuable insights but also highlights the need for further research to fully understand the capabilities and limitations of EUS-SWE.

### 3.2. Acute and Chronic Pancreatitis

Several studies have been published with promising results on the role of EUS-SWE in chronic pancreatitis. The initial study on this emerging topic was conducted by Yamashita et al., where they evaluated 52 patients who underwent EUS-SWE in 2018, of whom 16 had chronic pancreatitis [33]. The median Vs measurements using EUS-SWE for patients who had EUS features consistent with chronic pancreatitis, suggestive of chronic pancreatitis, indeterminate of chronic pancreatitis, and without chronic pancreatitis were 2.98 m/s, 2.95 m/s, 1.8 m/s, and 1.52 m/s, respectively. The authors reported that Vs measurements on EUS-SWE positively correlated with the number of EUS features indicative of chronic pancreatitis using the Rosemont criteria. Furthermore, the median Vs measurements were significantly higher in patients with EUS features consistent and suggestive of chronic pancreatitis compared to patients without chronic pancreatitis (*p*-Values < 0.001). The study reported an impressive area under the receiver operating characteristic curve (AUC) of 0.97 for diagnosing chronic pancreatitis, with 100% sensitivity and 94% specificity when using a Vs cutoff of 2.19 m/s. Additionally, the study also demonstrated a possible correlation between Vs measurements and pancreatic exocrine dysfunction and diabetes mellitus. These results highlight the promising role of EUS-SWE in identifying chronic pancreatitis. Building on that study, the same group conducted a prospective evaluation between 2019 and 2022, involving 49 patients who underwent EUS-SE and EUS-SWE to further evaluate their role in chronic pancreatitis [35]. The median Vs values utilizing EUS-SWE were significantly higher in patients with chronic pancreatitis (3.09 m/s) compared to patients without chronic pancreatitis (2.03 m/s) when using computed tomography as the standard diagnostic method (*p* < 0.001). Conversely, there was no significant difference when using EUS-SE between the two cohorts (chronic pancreatitis 3.29 vs. non-chronic pancreatitis 2.60; *p* = 0.64). The study showed that EUS-SWE Vs measurements were positively correlated with the severity of chronic pancreatitis based on the Rosemont criteria and the Japan Pancreatic Society criteria. On the other hand, this correlation was not seen when using EUS-SE. The analysis also demonstrated that EUS-SWE had superior accuracy compared to EUS-SE in diagnosing chronic pancreatitis, regardless of which diagnostic criteria were used. The areas under the receiver operating characteristic curve for EUS-SWE vs. EUS-SE were 0.77 vs. 0.61 when using computed tomography, 0.85 vs. 0.56 when using the Rosemont criteria, 0.83 vs. 0.53 when using the Japan Pancreatic Society criteria, and 0.78 vs. 0.61 when using exocrine dysfunction (All *p*-values < 0.001). Additionally, the authors highlighted that EUS-SWE measurements were positively correlated with EUS features of chronic pancreatitis, whereas this correlation was not evident with EUS-SE. These findings suggest that EUS-SWE might offer a more precise and reliable evaluation of pancreatic tissue changes associated with chronic pancreatitis.

A retrospective study by Shintani et al. on 50 patients, of whom 34 had chronic pancreatitis, conducted between 2020 and 2021, evaluated the diagnostic accuracy of EUS-SWE for chronic pancreatitis across different pancreatic regions: the head, body, and tail [36]. They showed that the most reliable measurements of EUS-SWE were noted in the body of the pancreas (93.5% of the measurements), followed by the head (91.6%), with the tail showing the least reliability (82.2%); *p* < 0.01. The study reported that Vs measurements were higher in patients with chronic pancreatitis at all three anatomical locations of the pancreas (*p* < 0.05). Specifically, the body of the pancreas had the highest diagnostic accuracy for chronic pancreatitis (AUC of 0.87 with a sensitivity of 87.5% and specificity of 82.4%) followed by the tail (AUC of 0.81 with a sensitivity of 87.5% and specificity of 73.3%), and the head (AUC of 0.79 with a sensitivity of 76.5% and specificity of 71%). The Vs cutoffs to diagnose chronic pancreatitis were 2.10 m/s, 2.33 m/s, and 2.22 m/s, in the head, body, and tail of the pancreas, respectively. In addition, the body of the pancreas demonstrated the strongest correlation between Vs measurements and EUS features of chronic pancreatitis according to the Rosemont criteria (spearman’s rank correlation coefficient of 0.55). In contrast, other research has indicated that the head of the pancreas may yield the most reliable measurements, with 85.1% of Vs measurements considered reliable compared to the body (75.5%) and tail (64.2%) [10]. This discrepancy highlights the need for further studies to determine which anatomical location in the pancreas provides the most reliable EUS-SWE measurements.

#### Bottom of Form

In addition to its application in chronic pancreatitis, existing literature suggests a potential role for EUS-SWE in autoimmune pancreatitis (AIP). Ohno et al. prospectively evaluated 160 patients who underwent EUS-SWE, including 14 with type 1 AIP, at a single center between 2016 and 2017 [32]. They showed that the median Vs measurement in patients with AIP at the body of the pancreas was significantly higher compared to normal controls (2.57 m/s vs. 1.89 m/s; *p* = 0.01). Furthermore, the study observed that among AIP patients who received steroid therapy (6 patients), the mean Vs value significantly decreased from 3.32 m/s to 2.46 m/s two weeks after the administration of steroid treatment. On the other hand, no correlation was noted between the EUS-SWE Vs measurement and serum IgG4 levels in patients with AIP. These findings indicate that EUS-SWE may be a valuable tool not only for diagnosing AIP but also for monitoring the effectiveness of treatment.

When evaluating acute pancreatitis, to the best of our knowledge, there are no data available assessing the role of EUS-SWE in this entity. Sezgin et al. conducted a prospective evaluation including 81 patients with acute pancreatitis and 78 controls who underwent transabdominal SWE measurement at a single center between 2019 and 2021 [37]. The study demonstrated that the mean EUS-SWE Vs measurement in AP was significantly higher than in controls with acute pancreatitis (10.97 vs. 7.72; *p* < 0.001). The authors also found that EUS-SWE Vs values in patients with acute pancreatitis, measured after clinical recovery, did not significantly differ from the Vs measurements taken one month later (8.96 vs. 8.83; *p* = 0.315) [37]. While this study suggests a potential role of SWE in evaluating acute pancreatitis, there remain clinical uncertainties about whether SWE measurements would be of diagnostic or prognostic value in acute pancreatitis. This may be due to the limitations of the transabdominal approach. To better investigate this potential, future studies utilizing EUS-SWE are warranted.

### 3.3. Fatty Pancreas

Fatty pancreas is a condition characterized by pancreatic steatosis and is commonly associated with metabolic syndrome, including obesity, hyperlipidemia, diabetes mellitus, and metabolic dysfunction-associated steatotic liver disease (MASLD) [38]. Fatty pancreas is diagnosed through imaging modalities such as EUS, computed tomography, or magnetic resonance imaging (MRI). A growing body of literature has shown the association of fatty pancreas with chronic pancreatitis and pancreatic cancer [39]. A prior study investigated histopathological examination of pancreatic cancer in a cohort of patients who underwent pancreatoduodenectomy and found significantly higher fatty infiltration in pancreatic cancer compared to the control cohort [40]. The authors also showed that fatty infiltration of the pancreas was associated with higher odds of having pancreatic cancer (adjusted odds ratio of 6.1). In addition, a prior prospective study including 9933 patients who underwent regular medical checkups demonstrated that patients with fatty pancreas are at a higher risk of developing chronic pancreatitis (adjusted odds ratio of 3.96) [41]. Therefore, it is crucial to enhance our understanding of fatty pancreas and improve diagnostic and prognostic evaluation methods. Given the established role of shear wave elastography in assessing tissue fibrosis, there might be a potential role of EUS-SWE in the evaluation of fatty pancreas. To investigate this question, a prospective study was conducted involving 167 patients who underwent EUS-SWE, with 38 of these patients diagnosed with fatty pancreas based on EUS features [42]. The median EUS-SWE Vs measurements in patients with fatty pancreas were significantly higher than in patients without fatty pancreas, and this was noted in the head (2.78 m/s vs. 2.29 m/s; *p* <0.01), body (2.56 m/s vs. 1.98 m/s; *p* < 0.01), and tail (2.69 m/s vs. 2.25 m/s; *p* = 0.01) of the pancreas. Furthermore, the elasticity was also significantly higher in patients with fatty pancreas compared to controls at the head (23.25 kp vs. 15.82 kp; *p* < 0.01), body (19.8 kp vs. 11.7 kp; *p* < 0.01), and tail (21.7 kp vs. 15.20 kp; *p* < 0.01) of the pancreas. The study also showed that SWE Vs measurements were independently associated with higher odds of fatty pancreas after adjusting for body mass index (BMI) and other demographic factors, including age, gender, race, alcohol use, and smoking history (adjusted odds ratio of 2.90; *p* = 0.04). In a follow-up study involving the same cohort, the authors evaluated 30 patients who underwent EUS-SWE and prior magnetic resonance imaging. The study demonstrated a positive correlation between the EUS-SWE median Vs measurement and the fat fraction determined by magnetic resonance imaging (Pearson coefficient of 0.42; *p* = 0.025) [43]. An increase of 1 m/s in Vs measurements on SWE-EUS was associated with a 6.4% increase in the fat fraction on MRI (*p* = 0.025). These findings, if externally validated, suggest that EUS-SWE has the potential to be a quantitative assessment tool for fatty pancreas.

## 4. Limitations of Endoscopic Ultrasound-Guided Shear Wave Elastography

Despite the growing body of literature and the emerging role of EUS-SWE in the evaluation of various pancreatic etiologies, there are several limitations of this technology which must be considered. These limitations can be broadly categorized into technical and clinical limitations. Technical limitations include the limited size of the ROI, which makes it difficult to delineate small lesions with a high degree of confidence. Furthermore, respiratory artifacts can introduce variability in SWE Vs measurements [29]. As for clinical limitations, they include the current uncertainties on the ability of EUS-SWE to differentiate benign from malignant pancreatic lesions [34]. Additionally, as noted above, prior data using transabdominal ultrasound SWE showed that Vs measurements significantly increased during acute pancreatitis episodes and remained elevated even after one month despite clinical improvement. This elevation of Vs might suggest persistent increased inflammatory processes in the pancreas, but it is unclear if this is a limitation of the technology or if it represents a clinically meaningful process [37]. However, the utilization of a transabdominal approach in that study makes it hard to draw any firm conclusions given the superiority of EUS in evaluating the pancreas. While EUS-SWE is still being evaluated as a promising new technology, its exact role at this time is yet to be definitively determined.

## 5. Future Directions

There are several avenues where EUS-SWE is being further investigated. These include both technical aspects and numerous clinical applications.

Current shear wave technology uses an acoustic radiation pulse force utilizing two-dimensional (2-D) imaging. While this provides valuable insight into the elastic mapping of the tissue being studied, new technologies are being investigated with the goal of improving this process. A prior study by Dong et al. proposed that introducing an advanced transducer array to implement 3-D imaging can improve the diagnostic accuracy of the tissue elasticity and offer a more comprehensive assessment of the mechanical properties of the tissue being studied [44]. The authors initially conducted an in vitro investigation and demonstrated that the proposed 3-D SWE model provided robust information on the elasticity of the tissue and could be cost-effective in clinical implications. Thereafter, they validated this technology in vivo on breast cancer tissue. While this is a promising modality, it requires validation in other organs including the pancreas. Other technologies that have been explored to improve the accuracy of SWE imaging include multiangle compound SWE that uses several push beams creating multiple shear waves [45], and Probe Oscillation SWE which generates a mechanical vibration from the transducer while simultaneously capturing parameters via a specialized mode “pulse-echo mode” [46]. While promising, these technologies have not yet been validated in the pancreas using EUS. Furthermore, implementing artificial intelligence in SWE technology may further augment its use in pancreatic disorders [47].

In addition to the technical aspects of EUS-SWE, future research is warranted to further evaluate its clinical role. This includes investigations into the role of EUS-SWE in the evaluation of the continuum of acute pancreatitis, recurrent acute pancreatitis, and chronic pancreatitis. Understanding its role in these clinical entities could provide deeper insights into diagnostic accuracy and outcome monitoring. Additionally, further studies are needed to elucidate the potential prognostic role of EUS-SWE in pancreatic cancer, which may include tracking changes in tumor stiffness over time or evaluating its response to treatment. Such information can enhance our understanding of tumor pathogenesis and help predict disease outcomes. Lastly, future research is needed to compare EUS-SWE Vs measurements between pancreatic cancer and pancreatitis, including acute, chronic, and autoimmune pancreatitis.

## 6. Conclusions

EUS-SWE is an emerging technology with promising results in the evaluation of various pancreatic diseases. Current literature provides strong evidence on the feasibility and safety of this technique [10,24,28]. Furthermore, existing data suggest that EUS-SWE is highly reproducible in the head, body, and tail of the pancreas [10]. There is growing evidence demonstrating the clinical utility of EUS-SWE in diagnosing and assessing the severity of chronic pancreatitis [35], AIP [32], and fatty pancreas [42,43]. While current data suggest EUS-SWE may have a limited role in differentiating different types of pancreatic tumors, future larger studies are warranted to further investigate the utility of EUS-SWE in predicting treatment outcomes.

## Data Availability

Data used in this study are available in the medical literature and can be found using the references provided.
